# Medico-Chirurgical Transactions

**Published:** 1817-01

**Authors:** 


### PART II.

### COMPREHENSIVE ANALYTICAL REVIEW OF MEDICAL LITERATURE.

"Tros, tyriusve, nobis nullo discrimine agetur."

*Medico-Chirurgical Transactions, Vol. VII. Part I. pp.* 300. 4 *plates,* 1816.

This tree of knowledge is rapidly extending its roots through every ramification of medical society, and thence extracting copious stores of valuable materials, that annually bloom, in luscious fruit, on its loaded branches. But it is confidently predicted, and openly avowed, that it will prove a *upas* tree to the Medical Journals; that, in short, like Aaron's serpent, it will swallow up the rest! For our own parts, we are free to confess, that we have long anticipated the storm that now bursts over the original departments of periodical Miscellanies; but, like British tars, we have viewed it undismayed, and prepared for the event.

The general exhaustion of society at large, and the pressure of the times, have extended their chilling influence to medical literature itself; and the diffusion of knowledge is checked by the expences which attend its march. There is at present a wall of brass between the upper and lower classes of the profession. The *former*, who have riches and leisure, monopolize the literary knowledge of the day; while the *latter* are left to starve on the scanty supply afforded by the analytical departments of a few Medical Journals. To remedy this *inequilibrium* in the balance of medical literature, this department of the MEDICO-CHIRURGICAL JOURNAL is constructed, so as to prove the GENERAL PRACTITIONER'S FRIEND, by bringing within his reach a vast store of information, which otherwise he might contemplate at a distance, but seldom receive.

We have been led to these reflections, by observing the expence which is unavoidably incurred by the country practitioner in participating the labours of this respectable society. To expend one guinea per annum on a single book, is more than many can do; and we shall not cease to deplore the policy which dictates the present mode of publication adopted by the Council; for, assuredly, it limits the advantages which would otherwise accrue to a very narrow circle.

But to proceed with the work before us.

ART. 1. *Chemical Analysis of the Mineral Waters of the Spa.* By ED. GODDEN JONES, M D.

We beg leave, in the name of the profession, to congratulate Dr. Jones on his *Asmodéan* escape from the *mystic phial,* and his re-appearance *at large* in the medical world once more! The first leap of the limping Devil was only to the steeple of a neighbouring church:—what a contrast does that of our countryman present! from Befort's boutique to the banks of the Rhine; from the thumb-bottle of Husson to the waters of Spa! Little as is the advantage which the profession at large can derive from the chemical researches of our author on the present occasion, it is greater than that which resulted from his former Essay on a "*Medicinal Water*," now famous for its power of curing *all diseases*, as well as gout, by a certain mysterious process termed death! We hope Dr. Jones has lived long enough to see the danger of lending a respectable name to the diffusion of a nostrum, and to regret that he should ever have been instrumental in recommending a drug, which, while it sheds a momentary balm over the *tortured limbs* of the sufferer, infuses a fatal poison that permeates through every avenue of the constitution!

Hinc subitæ mortes et intestata senectus!

To analyze an "Analysis" would be to compress water or expand steam; nor do we think that the generality of professional readers are much interested in ascertaining, to the twentieth part of a grain, how much iron or silex is contained in the Pouhon or Geronstere. Passing over 55 pages of tests and re-agents, therefore, we shall dwell a little on the medicinal properties of these mineral waters, which depend, we are informed, entirely on the iron and carbonic acid which they contain. The abundance of the latter modifies very much their effects as chalybeates, rendering them grateful and refreshing to the palate and stomach, exhilarating to the spirits, and tonic to the whole frame. They are therefore admissible in many diseases where simple chalybeates would be improper. These waters are also remarkable for their purity in other respects. Although they increase the secretions generally, they determine to the kidnies in particular, and to the skin. They are not directly purgative, but rather constipating. Their first sensible effects are vertigo and drowsiness; sometimes a kind of intoxication, followed by a strong disposition to sleep. This determination to the head is always abated when the urinary secretions are increased; and this is considered a good sign; hence the common inquiry, "*Si les eaux passent bien.*" The reverse is unfavourable, and demands a cessation from their employment. Dr. Jones agrees with Dr. Wall, in supposing that the effects of the Spa, as well as of other mineral waters, in causing vertigo, &c. may be owing "to the temporary plethora of the vessels of the head, occasioned by the facility and rapidity with which the pure fluid enters the absorbent system." This conjecture is strengthened by the disappearance of those symptoms on the increase of the urinary secretion. Mild purgatives are necessary when using the Spa waters: but the determination to the head is never to be overlooked in prescribing them. In certain habits it precludes their employment; in others, it renders venæsection an indispensible accompaniment. They allay thirst better than common water, "hence they are peculiarly grateful and refreshing in many cases of debility, attended with slow febrile symptoms, and a dry state of the tongue and throat." In affections of the kidnies and bladder they have been found highly useful; particularly where there is discharge of bloody, purulent, or fœtid urine, whether arising from ulceration or calculous concretions. Their good effects in these instances are probably owing to the rapidity with which they pass in large quantities through the urinary organs, by which they prevent the formation of fresh calculi, and wash away the small ones already formed. By cleansing the ulcerated surfaces, they also contribute to their healing. In these diseases, our author is of opinion, that their efficacy might be increased by the addition of a small proportion of carbonate of soda. As the Pouhon contains nearly the same quantity of iron as the Cheltenham water, an imitation of the latter is easily made by dissolving in the Pouhon a mixture of sulphas sodæ, sulph. magnes. and murias sodæ, in the proper proportions.

The people of Spa drink no other water at their meals. They are a robust, healthy race, and not liable to any predominant disease. These waters, in fine, are useful in various female complaints arising from relaxation and derangement in the uterine system; but in complaints of an inflammatory tendency, or accompanied by flushings of the face, full pulse, and determination to the head, they are, like other chalybeates, evidently improper. The duration of a course of these waters should be from six weeks to two months: longer continued, their stimulating effects begin to wear off. Exercise, cheerful society, and moderate amusements, are the auxiliaries at Spa, and contribute much to the efficacy of the waters. The pure mountain air, the amenity of the situation, which prompts the invalid to active and agreeable exercise, and the modes of living observed by the patients at Spa, are advantages which will render this beautiful spot a favourite place of resort for broken constitutions and valetudinaries of various descriptions.

Knowing as we do the talents and education of Dr. Jones, we hope to see them exercised in future on more important themes than — *les eaux medicinales.*

### Art. 2. *History of Two Cases of Angina Pectoris.* By Samuel Black, M.D. of Newry.

Of the various lesions to which the central organ of the circulation is subject, Angina Pectoris is not the least dangerous or distressing. To be charged with the distribution of vital fluid to every other part of the body, while itself cannot participate, is, like Tantalus, to be surrounded with drink, but unable to quench the thirst! Such is the situation of the heart in this dreadful disease; and he who, like ourselves, has seen the unhappy sufferer die many deaths in it, can never have the gloomy picture erased from his mind!

It is known that Dr. Black communicated two cases of this disease to the medical world through the medium of the Memoirs of the Medical Society, vol. iv. and vol. vi. Since that time the pathology of the disease has been better understood, though physicians are not yet unanimous respecting it. Dr. Parry, of Bath, has, indeed, concentrated into one luminous point of view the scattered rays that flowed from a variety of sources, in his learned and truly didactic work on that subject; yet still a discrepancy prevails.

*Case* 1. Mr. Marron, an eminent school-master, had been through life a healthy man, not subject to gout or any other disease. He had, however, by a consecutive train of calamities, experienced the most bitter domestic affliction. His disease commenced in 1799, with an intolerable sense of anguish under the sternum, especially when walking up an ascent, or at an accelerated pace, accompanied by a severe pain, diffusing itself from the chest to the left scapula, and down the left arm to near the elbow. The impression was, as usual, that another step, and life would be extinguished. On standing still, these symptoms vanished. At the end of eighteen months, nocturnal paroxysms began to break his rest, and they were alleviated by opiates and cordials. In 1803, symptoms of hydrothorax were superadded. These were repeatedly relieved by digitalis, squill, and the quicksilver pill; but uniformly recurred after a short interval. He expired suddenly in July 1804.

*Sectio Cadaveris.* The cellular membrane loaded with fat; the cartilages of the ribs ossified. In the bags of the pleura a large quantity of water. The heart was loaded with fat, large, flabby, and soft. The valves were sound. Several osseous scales on the internal surface of the aorta, near its origin. The coronary arteries were ossified throughout their whole extent.

*Case* 2. The Rev. J. M'Cormick had been occasionally affected with gout, but neither frequently nor severely. He had long had hæmorrhoidal discharges, without pain or external tumour. These had ceased three years, and he had become fat and plethoric. His habits were regular and temperate. Dr. B. visited him in January 1815.

He had experienced much fatigue of body and mental emotion in the Christmas week preceding; after which he was suddenly seized, while walking up an ascent, with a feeling of exquisite distress in the chest, accompanied by such a sense of debility and sinking, as appeared to threaten instantaneous dissolution. These vanished on standing still, but were repeatedly renewed on attempting to walk. These paroxysms were easily renewed ever afterwards, by any slight muscular exertion, and were uniformly accompanied by a very painful sensation, to which he applied the term *scalding*, diffusing itself from the left side of the thorax towards the scapula, the œsophagus, and down the left arm to the insertion of the deltoid muscle, where it terminated. On minute inquiry, Dr. B. found that the patient had been occasionally affected with feelings of the same kind for three or four years, but never assuming such an exquisite form. His pulse was from 50 to 56, and weak. "In the month of February, he began to be attacked out of sleep by the nightly paroxysms." His feelings on these occasions were "as if every thing within were at a pause, or if he were just going to die." He could sometimes baffle the paroxysm by concentrating his attention on some interesting kind of reading. He also found relief from a moderately full inspiration and expansion of the chest. Food increased the painful symptoms. In March, hydrothorax was developed, and the ankles became œdematous. These symptoms were relieved for a time by digitalis and blue pill; but they soon returned. By the end of June, the cellular membrane was universally loaded with fluid, and the symptoms of hydrothorax so urgent, that he could rarely lie down. He expired in July.

*Sectio Cadaveris.* The cellular membrane excessively loaded with fat; cartilages of the ribs ossified: eight or nine pints of water in the thorax; none in the pericardium. Heart loaded with fat; large, flaccid, tender, and pale. Several yellow spots on the surface, but soft. Valves sound.

"The coronary arteries were ossified in a remarkable manner. They are in my possession. One of them divides, immediately after its origin, into three principal branches, every one of which is osseous through its whole extent; saving that the bony structure is interrupted at intervals. The longest branch, which is five inches long, is not pervious to the smallest probe for more than half an inch; the other two branches not so far. The other coronary does not divide into branches, but its calibre is perfectly obliterated, and they are all as rigid and incompressible as any other bone of the same diameter. About three inches of the aorta being cut out, its internal surface exhibits a number of osseous scales, surrounding more especially the origin of the coronaries. There is one very remarkable lamina of bone, one-third of an inch long, nearly as broad and as thick as a shilling." p. 77.

Of the treatment Dr. Black thinks it needless to speak, but refers to Drs. Parry and Blackall for the best information on this head. We shall abridge some of his general remarks.—1st. Of the four patients which Dr. B. has seen, one only had been liable to gout. 2d. Two terminated by thoracic effusion; one by pericardiac effusion; one without effusion. 3d. Ossification, more or less complete, of the cartilages of the ribs was found in all. 4th. The disease appears to be connected with a full habit, and accumulation of fat: it may, in some instances, have connexion with suppressed discharges. 5th. Issues seem advantageous. 6th. Ossification of the coronary arteries appears to be the cause, not the consequence, of the disease. 7th. Dr. B. thinks Dr. Parry right in classing the disease under the genus Syncope, " as the paroxysm seems to consist very much in an impediment or suspension of the vital action of the heart." 8th. Is a virtual repetition of 6. 9th. Mental emotion appears to be a powerful predisposing cause. 10th. The female sex seem exempt from the disease. 11th. Since syncope angens appears to have some relation to obesity and a plethoric habit; since it generally happens among those who sit down to a plentiful table; since every thing that hurries the circulation, or urges the machinery by which that function is carried on, beyond its capacity of performance, seems to excite the paroxysm; since medicine seems to offer little relief; " would it be irrational to propose the trial of a regimen as low and abstemious as is compatible with life?" Such trial should be made on the very first threatening of the disease, for if that be completely established—

——— Sero medicina paratur
Cum mala per longas invaluêre moras!

We shall defer any remarks on this subject for the present, as an interesting Case of the disease, with practical observations, will shortly appear in this Journal, by one of the Editors.

ART.

Art. 3. *Account of a very fatal Affection of the Pudendum of Female Children.* By Kinder Wood, Esq. Surgeon, Oldham.

There are many diseases which were once termed *rare*, because they had not previously attracted notice. We may instance that which has been described in the preceding article. Forty or fifty years ago, the very name of the disease was unknown; but now, hardly a practitioner of eminence has failed to meet with it at one period or other of his practice. We cannot suppose that angina pectoris did not exist, as well in the days of Hippocrates as in those of Heberden. We are therefore always ready to give record to apparently new and singular disorders, confident that too little attention has been paid to the *less usual* aberrations from health, from the idea that such might never again be presented to the practitioner's notice, and consequently that it would be a waste of time to describe or remember them.

Of the affection under consideration, Mr. W. has seen twelve cases, all occurring between one and six years of age. Two only recovered. It commences with alternate chills and flushes; slight head-ache, dulness, nausea, loss of appetite, thirst, clay-coloured tongue, torpid bowels, languor, listlessness. These precede the local affection about three days. Then comes pain in voiding urine; or, in young children, cries and struggles in that act. One or both labia pudendi will now be found inflamed and enlarged; the inflammation having a dark tint, and soon extending over the clitoris, nymphæ, and hymen. In twenty-four hours, a crop of small vesications, internally and externally, burst, and quickly coalescing, form larger ulcers. The secretions from these, and from the vagina, are dark coloured, and peculiarly offensive; they are copious, and, by irritating the adjacent parts, extend the disease along the perinæum to the anus and neighbouring regions. The pulse is now quick and irritable; the face becomes of a peculiar pallid hue; the skin singularly white. Opening medicines bring away dark, slimy, and offensive stools. The bottoms of the ulcerations are, in some places, foul and deep; in others, superficial, and sprinkled with small red granulations. The pain is so great during any motion of the pelvis, that children old enough to consult their own ease, constantly lie upon their backs with the knees bent and thrown open. Retention of urine occasionally attends, and should be guarded against. The course of this disease is various and uncertain.

"From the time that the ulcerative action is completely established, the enlarged labia diminish, and the redness disappears, the ulcer successively extending over parts which had been inflamed. The character of the disease at this time is that of a deep, foul, and spreading ulcer, upon parts weakened by a peculiar inflammation, and a constitution weakened and injured by previous febrile symptoms. The external organs of generation are now progressively destroyed; the peculiar pallor of countenance increases; the pulse becomes quick and weak; the appetite fails; the bowels become loose; the skin of the thighs hangs loose and flabby as in marasmus; the discharge from the parts increases, and becomes more and more offensive, till the patient is worn out and expires." p. 88.

*Treatment.* The bowels are to be cleared by sub. hydr. and pulv. rhei, which uniformly bring away dark-coloured and offensive stools. The affected parts should be frequently washed with the liq. plumb. acet. dilut. slightly aired, and poultices, made with the same liquor and soft bread, applied warm immediately after the parts have been washed. The decoct. cinchonæ must be commenced so soon as the ulcerative action is begun; to which may be added, the confect. aromat. tinct. columb. and small doses of tinct. opii, opening the bowels every second or third day. A moderate use of Port wine may be allowed. When, in the latter stages, the bowels become loose, the elect. mimos. catechu is of great service, increasing the tinct. opii. Mr. W. has given the bark in substance in this disease with good effect.

There is one point of view in which the consideration of this disease is highly important; namely, in a forensic. There is little doubt but the affection in question has been frequently considered in court as evidence of violence and venereal infection; inflammation, ulceration, and discharge, having always had particular attention in a consideration of the evidence. Dr. Perceval, in his Medical Ethics, relates a case, where, from his statement, a coroner's jury brought a verdict of murder against a boy; but, before the trial was brought on, several other and similar cases occurred that convinced him he had given a wrong evidence, and the boy was accordingly acquitted. Mr. Wood appears to be an intelligent and observant practitioner.

ART.

ART. 4. *Case of ununited Fracture of the Os Humeri, treated successfully by the Seton.* By JOSIAS STANSFIELD, Esq. Surgeon to the Leeds Infirmary.

A strong healthy man had his left arm fractured by a fall from a coach, in December 1814. Although the fracture was attended to by a surgeon, no union took place, and the patient's arm continued in a useless state to July 1815, when he was admitted into the Leeds Infirmary.

The two portions of bone could be made to form a very considerable angle, and he was unable to raise the arm from the side. The fracture was very oblique, occupying nearly four inches, from the insertion of the deltoid downwards.

"I commenced the operation, by making a division of the integuments, an inch and a half long, on either side of the bone, about the centre of the fracture; the biceps muscle was then drawn inwards, and the cellular membrane divided, down to the sulcus; on the outer side I was obliged to divide some fibres of the triceps muscle; a curved seton needle was now easily pushed betwixt the ends, the intervening substance being very soft. The seton string consisted of a skein of silk, doubled, and well waxed. The lips of the wounds were drawn together by strips of adhesive plaster, and a compress and bandage applied. On the following day, the patient was in very great pain, and had a smart attack of fever, which induced me to take off the dressings, and apply a bread poultice, adopting at the same time the antiphlogistic regimen. This plan was continued till the inflammation had subsided, and suppuration was completely established. During the first fortnight, the slightest motion of the arm gave him very acute pain; but by the 16th of August being comparatively easy, I dressed the wounds with ointment and lint, and applied a compress and splints, supporting the whole by two leather straps; his arm was allowed to hang by his side, by which it was kept straight, and this position was the easiest to him." p. 105.

He was now ordered to get up daily, and walk out in the open air, to have as much meat and porter as agreed with him, and to take the decoction of bark with sulphuric acid. The wounds were dressed daily, the seton was moved backwards and forwards several times at each dressing, and by the 26th of August the arm had gained some strength. On the 8th of September, the seton string was reduced one half; after which, he experienced no uneasiness. The seton was withdrawn on the 3d of October, and he soon regained the use of his arm.

ART. 5. *Case of Wound in the Face, requiring the Operation of tying the Carotid Artery.* By CHARLES COLLIER, Esq. Surgeon to the Forces.

A drummer of the 44th regiment, aged 20, was wounded by a spear or sword, on the 17th of June, the instrument passing in at the angle of the left jaw, penetrating the mouth, and severely lacerating the tongue. Till the 22d, nothing remarkable occurred after the primary hæmorrhage. On that evening, arterial blood jetted up with force from the bottom of a narrow deep wound, flowing in various directions, as if from several branches of the external carotid. Dilatation was attempted, but the bleeding vessels could not be traced. Compression produced only a temporary mitigation of the hæmorrhage which flowed through the mouth, as well as through the external wound. The operation was then determined on.

"The patient being laid on a table, and his neck a little on the stretch, I made an incision of rather better than two inches, on the inner and lower surface of the sternal section of the sterno-cleido, dissected off the platonic* and cellular substance, and detached from its border the thyroid vein, after having exposed it. The dissected muscle was held aside by Mr. Cooper, while I detached the jugular vein from surrounding connections, and took off so much of its cellular covering as should leave sufficient indication of its coat: this difficulty removed, I laid open the sheath of the artery, pushed aside the par vagum, and a little dissection enabled me to pass a blunt probe, armed with a ligature of two threads, round it. The vessel was then secured at about three-fourths of an inch from the sternum, and the wound approximated by two interrupted sutures. The operation occupied nearly an hour; delayed, in some degree, by its being performed by candle-light, and by the necessity we were under of raising the patient up, occasionally, to clear his mouth of coagula; he lost no blood from the operation; the hæmorrhage ceased the instant the ligature was fastened." P. 109.

There was no return of hæmorrhage. On the 5th of July, the ligature came away, and he was discharged cured on the 12th of August.

ART. 6. *History of a Tumour successfully removed from the Face and Neck, by previously tying the Carotid Artery.* By WILLIAM GOODLAD, Esq. Surgeon, Bury.

THIS we consider to have been a most daring operation, and one that stamps a character of intrepidity and deci-

* We should suppose he means the platysma myoides.

sion on the operator. The patient was a middle-aged woman, upon whose case a consultation had been held a few days previously at Manchester; the result of which was, that no operation was advisable.

" She had a large tumour extending from near the external canthus of the left eye, down the cheek to the infra-orbitary foramen and the root of the ear, which was elevated by the tumour, and passing under it, it extended behind the mastoid process. Anteriorly, it extended to the chin, and to the trachea, which it partially covered, and hung pendulous over the clavicle. The circumference of the base of the tumour, when last measured, was twenty inches; since which period it had increased rapidly, but I regret not having ascertained its exact size: it must, however, from the space it occupied, have been at least twenty-eight inches.

" In the upper portion, comprised between the corner of the os hyoides upwards to above the zygoma, the tumour in the base was as large as, or larger than in its middle or apex, but below this point, its attachment to the neck was less extensive; and though hanging pendulous over the clavicle, its lowest connection with the neck was three quarters of an inch distant from that bone. On elevating the tumour, in this lower space, with a hand on each side, and passing the fingers at the same time under it, it might be clearly ascertained, that there was no connection with the vessels; but, as it hung over the trachea, and was connected with it, it required a very careful examination, to be convinced that they were not united. The corner of the os hyoides was, however, moveable under the tumour; and independent of it, respiration was tolerably free, when in an erect posture, and on passing the fingers forcibly between these parts, I was pretty well convinced that they might be separated. The œsophagus here was too deep to be implicated in the disease.

" Above the corner of the hyoid bone, the base of the tumour was very extensive and deep, impeding deglutition considerably. On directing the finger, passed into the mouth, towards the base of the ramus and angle of the maxillary bone, and into the fauces; the intervening substance appeared considerable, and authorized an opinion that the fauces would not be cut into, nor indeed exposed, by its removal. The submaxillary gland was pressed inwards, but did not appear enlarged or hardened, and the membrane of the cheek was also healthy, though the tumour appeared in contact with it.

" The disease commenced behind the angle of the jaw, and now extended behind the mastoid process, and was so closely connected with the subjacent parts, that the finger could not be passed under it. Its connections here, therefore, were only to be ascertained by collateral circumstances; and as the whole site of the parotid gland was coveied by it, it became a question worthy of serious consideration, how far that substance was enveloped in the disease, particularly as it had been stated, on most respectable authority, that its removal was impracticable. The patient's being able to open the mouth and masticate, was a complete proof, that if the whole substance of the gland were diseased, it had become dragged from its situation by the weight of the tumour, and that the tumour itself did not dip into the fossa behind it. Yet, every other circumstance rendered it certain that the parotid gland was enveloped in the disease, which was, indeed, verified in the operation. But the extent of the disease here became less important, if the carotid artery were previously secured, as I intended. I ought to have observed, that the tumour was perfectly moveable, though its motions were very limited, and that it had, therefore, no bony attachment either to the jaw or to the zygoma.

"The surface of the tumour was divided into large tuberculi, and the apex of each tubercle was rendered more prominent by a collection of fluid: it was fleshy, but had neither the hardness nor any other external character of carcinoma. There was no absorbent gland affected in its neighbourhood, and though ulcerated in two points, one of which was extensive, the aspect of the ulcer was not forbidding, being partially granulating, and partly sloughing; fungus hæmatodes was, therefore, not to be dreaded. Yet, a discouraging circumstance arose from a Charlatan's having, as he believed, succeeded in removing the complaint by the knife, at an early period of its history, on which occasion the hæmorrhage was very alarming. After a short time, the disease re-appeared, and had only been nine months in attaining its present enormous size. Large varicose veins spread over the surface, and as the skin was uniformly diseased, ulceration might be expected to extend to them, and hæmorrhage to be an inevitable consequence.

"The patient being previously laid upon a table, with her head as low as she could bear, from the danger of suffocation by the pressure of the tumour, an incision about four inches in length was made through the integuments, and the tumour at the same time brought as much as possible from over the trachea, to reach the head of the sterno-mastoid muscle; the course of which could not previously be traced, the sternal edge of its tendinous insertion being only just perceptible; blood followed the incision very profusely: but having dissected the sac of the tumour from the neighbouring integument, the inner edge of the muscle came into view, and the platisma myoides being more freely divided, the artery was plainly felt beating at the bottom of the wound. The knife was now laid aside, the cellular membrane separated by the fingers, the arterial sheath exposed, and the vessels secured between the finger and thumb. I endeavoured to separate the fibres of the fascia by the nail of the fore-finger and thumb, so as to pass the finger beneath the artery, and by its constant apposition with the vessel, prevent any other part from being included. The resistance to this attempt was great, and the eyed-end of a probe, which I had formerly found useful, was directed to the outside of the vessel, so as to press against the finger on the opposite side; but though bent at different angles to accommodate it to the wound, the shaft of the strongest probe was too slender to be directed with accuracy to so great a depth, and generally turned in the wound. The size of the tumour very much added to the difficulties of this stage of the operation, not only by rendering the wound deeper, but as it was necessary to keep it aside, and this necessarily impeded the fingers; whilst, if left at liberty, by pressing upon the probe, it changed its direction. The point of an aneurismal needle was next tried with no better success, but on directing the shaft of the needle into the wound, it was pressed against the finger with great facility; and when convinced that no part intervened, for the vessel had been stripped of its fascia, except at its posterior part, where, on insinuating it between the thumb and finger, passed on each side, only a few fibres were left undivided, and the division of these being accomplished, the finger was gradually and cautiously passed under the vessel, with the needle in contact with it, passed from without inwards. But it had to be turned, which, in so deep a wound, was with difficulty accomplished; and though the greatest care was observed, by keeping the finger of the left hand between the end of the instrument and the trachea, and pressing at the extremities of the instrument, to bend and accommodate it to the cavity, more violence was done to the vessel than was desirable.

"The patient bore this tedious operation with great fortitude, only endeavouring to relieve the irritation which the fingers produced, by a frequent attempt to swallow. A considerable quantity of blood was already lost, and the ligature therefore was immediately tied as low as possible in the wound. She complained of much pain, when the thread was drawn tight, extending from the wound to the whole of that side of the head.

"The fluid contained in the apex of each tubercle was next discharged, to reduce the size of the tumour, and render it more easily handled, as well as to lessen the pressure on the trachea, of which she complained heavily. The incision being then carried along the base of the tumour to its upper part, it was dissected from the cheek; though, during this, and the remaining part of the operation, the fingers were used wherever practicable; but occasionally stronger ligamentous bands rendered division by the knife necessary. The hæmorrhage, on dividing the integument, which connected the upper portion of the tumour to the head, where the external veins were ramified over it, was considerable; and on dissecting downwards, the ramus of the jaw became cognizable: and in this part, as well as under and behind the ear, each stroke of the knife was followed by a gush of blood, and occasionally in a tremendous stream; which, after continuing for a few seconds, ceased. The patient was very faint when the operation was finished, but a little wine at intervals soon restored her to complete perception.

" Besides a general oozing, there were a few points from which blood flowed rather freely, though the blood was venous; and as a matter of caution rather than of necessity, they were secured by ligature. The wound now exhibited the following appearances: the whole sterno-mastoid muscle was exposed, and its fibres dissected clean, except about half an inch from its insertion into the clavicle. The wound extended backwards from behind the mastoid process to the trachea anteriorly, but became narrowed in the direction of the muscle at the lower part of the neck. The submaxillary gland was exposed, and about one fifth of its substance, not appearing healthy, was removed. The digastric and the greater portion of the mylo hyoideus were exposed, the ramus of the jaw was only covered by periosteum, except where covered by the masseter muscle; part of which, not appearing healthy, was dissected away. The whole of the condyloid process of that bone was laid bare in the same manner, and behind it the pterygoid muscles were also exposed. The membrane of the cheek was only covered by a cellular substance, which did not appear healthy; but sufficient skin was saved to cover the zygoma. The parotid gland was entirely removed." P. 123. The patient recovered.

We thought it an injustice to Mr. Goodlad to abbreviate the description of the tumour, or the steps of the operation, and we have therefore given them both in his own words. They do him infinite credit.

In our analysis of the first half of this volume, we have allotted each article a space rather proportioned to its utility than its learning or splendour—a standing maxim in the Medico-Chirurgical Journal. There may be infinitely more eclat and science in the construction of an air balloon, than in that of a hackney coach; yet, for the *common purposes* of life, there are few who would not prefer the latter to the former vehicle.

( To be continued. )

